# Effect of hip CPM on gross motor function and development of the hip joint: a single-center randomized controlled study on spastic cerebral palsy children with hip dysplasia

**DOI:** 10.3389/fped.2023.1090919

**Published:** 2023-05-09

**Authors:** Lulu Wang, Nuochen Zhang, Liwei Fang, Zhenzhen Cui, Huihui Niu, Fuli Lv, Dayong Hu, De Wu

**Affiliations:** ^1^ Pediatric Neurological Rehabilitation Center, Pediatric Department, The First Affiliated Hospital of Anhui Medical University, Hefei, China; ^2^ Department of Pediatrics, Anhui Hefei Southeast Surgical Hospital

**Keywords:** hip dysplasia, hip continuous passive motion, goal-directed, hCPM—hip continuous passive motion, GMFM—gross motor function measure, MP—migration percentage, AI—acetabular index, HHS—Harris hip functional score

## Abstract

**Objective:**

To investigate the effectiveness of hip continuous passive motion (hCPM) on hip development at skeletal maturity and gross motor function for spastic cerebral palsy children with hip dysplasia.

**Methods:**

Prospective case–control research of hCPM with goal-directed training versus merely goal-directed training. On the basis of goal-directed training, the hCPM group used the hip joint CPM instrument (the external fixator was connected to the power device to make the hip joint carry out continuous passive movement) for 40–60 min, twice a day, and five times a week, and received continuous training for 8 weeks simultaneously. The control group received only goal-directed training for 8 weeks. Functional outcomes pertaining to the affected hip joints were assessed via gross motor function measure (GMFM), migration percentage (MP), acetabular index (AI), and Harris hip functional score (HHS) at the time of enrollment and the end of the intervention.

**Results:**

The case–control research included 65 participants (mean age = 46.20 months, SD = 17.09 months; Gross Motor Function Grading System level: III = 41, IV = 24) who were randomly selected to either the hCPM (*n* = 45) or the control group (*n* = 20). No differences were found in baseline (acquisition phase) GMFM, MP, AI, or HHS (*t* = −1.720, *P* = 0.090; t* = 1.836, P* = 0.071; *t*# = −1.517, *P*# = 0.139; t* = −1.310, P* = 0.195; *t*# = −1.084, *P*# = 0.097; *t* = −1.041, *P* = 0.301). At the 8-week follow-up, GMFM, MP, AI, and HHS significantly improved over baseline in the hCPM group (hCPM group: *t* = 18.59, 20.172*, 40.291#, 16.820*, 32.900#, 28.081; *P* < 0.001). Between-group differences at 8-week follow-up times points favored the hCPM group for GMFM (*t* = −2.637, *P* = 0.011), MP (t* = 2.615, P* = 0.014; *t*# = 3.000, *P*# = 0.006), AI (t* = 2.055, P* = 0.044; *t*# = 2.223, *P*# = 0.030), HHS (*t* = −4.685, *P* < 0.001) (*: left side; #: right side).

**Conclusion:**

Spastic cerebral palsy children with hip dysplasia achieved meaningful functional improvement after 8 weeks of goal-directed training with hCPM therapy.

## 1. Introduction

Cerebral palsy (CP) is a permanent central motor and postural dysplasia and mobility limitation syndrome produced by non-progressive brain injury in the growing fetus or infants (1). It is a life-long disabling disease that seriously endangers children’s health and causes serious burden to society and families. Other serious consequences such as epilepsy, intellectual disability, visual and auditory impairment, malnutrition, sleep disturbance, and secondary skeletal and muscle distortion are commonly associated with the disease (2).

The clinical classification diagnosis of cerebral palsy can be further divided into spasticity, dyskinesia, ataxia, or other and mixed types according to the nature of the motor disorders caused (3). Among them, spastic cerebral palsy is the most common type of movement disorder, affecting about 80% of children with cerebral palsy (4).

The hip joint (HJ) is recognized as the largest joint in the human skeletal system, and it is also the joint that causes the most problems and troubles for children with cerebral palsy from the perspective of motor function in sitting upright, lying down, and walking. The buttocks of CP children tend to be close to normal at birth. During the growth and development process after birth, due to the influence of abnormal traction caused by cerebral palsy, a series of problems gradually emerge as the children’s bones grow up and mature, resulting in acetabular dysplasia, or even the relative abnormality caused by a deviation of the femoral head and acetabulum in the spatial position. In other words, the femoral head is partially or completely dislocated from the acetabulum until serious or even irreversible consequences occur (5).

Hip dysplasia is closely related to age and can be measured by using the Gross Motor Function Grading System (GMFCS) in children with cerebral palsy (6). For children with GMFCS grade II or above, the rate of incidence of hip anomalies can range from 40% to 70%, including joint dysplasia, subluxation, or complete dislocation. Hip dysplasia is primarily responsible for slowing down the motor development of children with cerebral palsy, especially walking ability (7, 8). Although non-operative interventions (such as postural sleeping systems, seating modifications, abduction bracing, and injection of neurolytic agents and so on) have attempted to reduce or prevent hip displacement, the guidelines only recommend regular monitoring and timely operation for abnormal hip joint problems in children with cerebral palsy (9, 10).

Current research suggests that botulinum toxin and soft tissue repair surgery can help eliminate the need for future surgery, but when hip joint subluxation or dislocation is evident, successful reduction can be achieved through surgery. As to whether the patient can recover to the preonset state of motion after reduction remains an unanswered question. In traditional rehabilitation treatment measures, the technique of slow and continuous stretching is often used to prevent muscle contractures and minimize spastic and tonic states caused by cerebral palsy, a fundamental disease. However, passive stretching requires a large number of therapists and can be sustained only for a relatively short period of time.

In recent years, continuous passive movement (CPM) in orthopedics has gradually caught the popular imagination. CPM refers to the continuous passive movement of a joint assisted by some mechanical instrument. The device of continuous passive movement provides repeated training and uses less manpower to input the repeated stretching, in order to achieve the effects of promoting blood circulation and reducing limb swelling and exudation. The aim is to prevent prolonged muscle spasm and joint adhesion and stiffness. At the same time, continuous passive movement causes no damage to the normally active articular cartilage, which can promote a rapid and complete healing of the full-thickness defect of the articular cartilage, while preventing the necrosis of the femoral head.

Inspired by this finding, in 2017, the research team collaborated with Hefei Huijia Medical Technology Co., Ltd. to develop a continuous passive motion instrument (ZL201220503585.5) (11) for treating developmental dysplasia of the hip (DDH). It uses external forces to carry out continuous slow, continuous, and uniform passive motion of the lower limbs on both sides. In theory, this repetitive activity can promote the recovery of blood circulation and related motor nerve function, improve the excitability of nerve activity, establish a benign circulation, and promote the recovery of the skeletal muscle to normal condition (12).

The answer to the question whether this special strategy of continuous passive hip movement can effectively inhibit the development of hip dysplasia in children with cerebral palsy can be provided by performing a clinical evaluation. In order to solve the problem of the lack of early intervention methods for the development of the hip joint in children with severe cerebral palsy, the research group plans to use the hip CPM instrument to conduct a prospective study on children with spastic cerebral palsy with dysplasia of the hip joint.

The purpose is to observe the impact of the hip CPM instrument on the gross motor function and the development of the hip joint in children with cerebral palsy and to provide a basis of reference for multicenter and large-sample studies.

## 2. Methods

### 2.1. Participants

This prospective RCT informed all participants (including patients and their parents), and their consent was obtained in writing. The registration number for this RCT in the Chinese Clinical Trial Registry (ChiCTR) is ChiCTR2000040948. Our study was approved by the Clinical Medical Research Ethics Committee of the First Affiliated Hospital of Anhui Medical University.

A total of 65 children were eligible for admission and were able to fully cooperate with the treatment, of which 45 were randomly divided into the hip continuous passive motion (hCPM) group and 20 into the control group. According to the single-blind principle, the professional evaluator will evaluate the gross motor function of children with cerebral palsy and the structure and function of the hip joint development. The professional pediatric neurologic rehabilitation physician will conduct the safety inspection and develop the rehabilitation treatment plan for these children.

**Figure 1:**
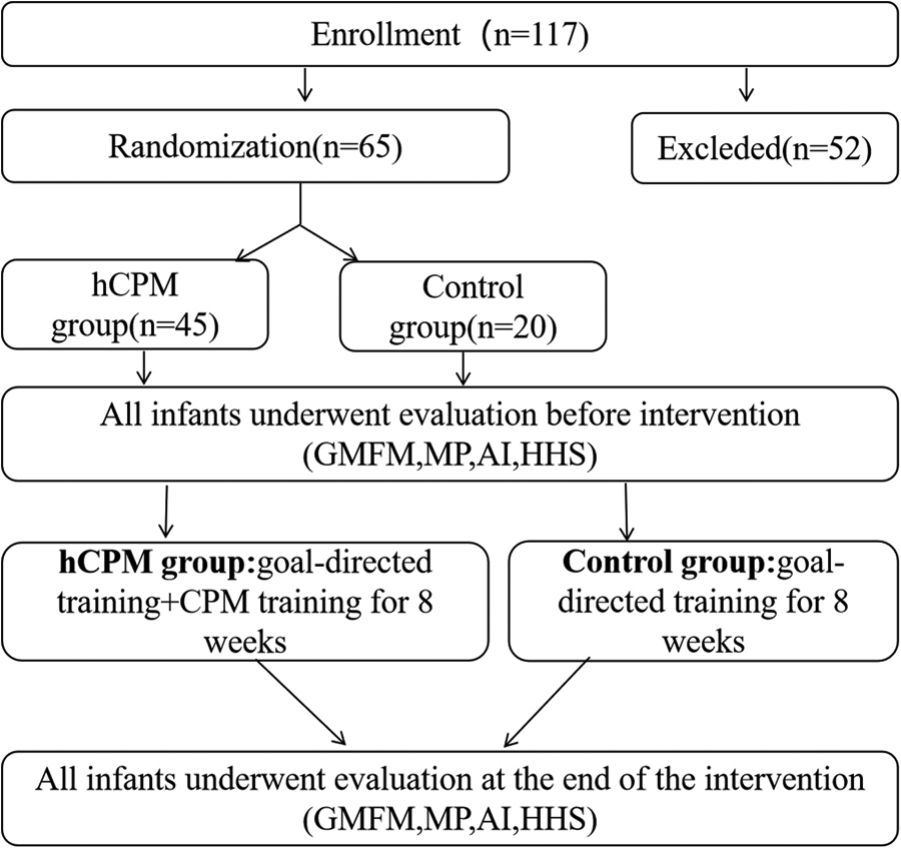
Consort flow diagram showing the flow of participants through the trial.

The whole recruitment process and study flow chart are presented in Figure 1.

### 2.2. Study design

Two attending doctors conducted a preliminary evaluation of the children simultaneously. A total of 117 children with spastic cerebral palsy who were admitted to the pediatric neurology rehabilitation center of the high-tech zone of our hospital during the period between June 2020 and December 2021 were selected for the study, of whom 65 children met the preliminary criteria for inclusion in this study.

The inclusion criteria were as follows: (1) Children with a definite diagnosis of spastic cerebral palsy, with the plain film of the pelvis suggesting a hip dysplasia; (2) Children with age ranging from 2 to 6 years old, with no gender limitations; (3) Those having clinical manifestations of spastic paralysis accompanied by hip dysplasia; (4) Those with a GMFCS grading of III–IV; (5) Those who sought to voluntarily participate in this study and signed informed consent on their own or obtained signatures from their legal representatives.

The exclusion criteria were as follows: (1) Children who had fracture, abnormal bone density, and high muscle tone; (2) Children with local skin infection or open wound; (3) Those who had participated in clinical studies/research in the last 3 months before their enrollment in this study; (4) Other types of clinical evidence showing that a particular patient is not eligible for a study of this kind.

### 2.3. Intervention

#### 2.3.1. Control group

Children belonging to the control group received the same goal-directed training plan as that of the hCPM group. This plan included the following: physical therapy, hand function therapy, speech therapy, guided educational therapy, and physical factor therapy (once a day, five times a week, between 25 and 35 min each time for 8 weeks).

#### 2.3.2. Goal-directed training

Taking the expected motor function index as the phased goal throughout the whole process of rehabilitation training, the motor function of children with cerebral palsy can be improved (13): it can help improve the active motor performance of children with cerebral palsy and guide them to generate active movement, thus enabling them to complete the tasks and activity goals in daily life. The training includes the following five items:

(1) Physical therapy: This includes muscle strength training, motor learning, and task-oriented training of the major muscles of the limbs, aimed at improving the gross motor function and perceptual motor ability of the target task. Each training should be maintained between 25 and 35 min, once a day, and five times a week, and continuous treatment should be provided for 8 weeks.
(2) Occupational therapy: The aim of this therapy is to design targeted work activities for each transposition to train the children’s sense of perception, hand-eye coordination ability, fine function of the upper limbs and coordination ability of both hands, and improve the fine activity of the upper limbs and hands. Each training should be maintained between 25 and 35 min, once a day, five times a week, and continuous treatment must be given for 8 weeks.
(3) Speech therapy: This is in the form of a one-to-one therapy. The speech therapist will carry out respiratory function training, oral muscle group control ability training, cognitive function training, and language function training for children to promote their speech expression and intellectual development. Each training should be maintained between 25 and 35 min, once a day, and five times a week, and continuous treatment must be initiated for 8 weeks.
(4) Guided educational therapy: Rehabilitation therapists carry out diversified rehabilitation training by way of education and teaching, adopt the operation mode of group class, maximize the potential of children's independent movement by continuously providing scientific guidance skills and commands through a guide, stimulate the awareness of active participation, and improve the children's sports, language, cognitive, and other functions. Each training should be maintained between 25 and 35 min, once a day, and five times a week, and there should be continuous treatment for 8 weeks.
(5) Physical factor therapy: This includes the use of physical instruments such as transcranial magnetic circulation, transcranial ultrasound, hydrotherapy, electromyography biofeedback, and muscle excitation to assist in treatment, aimed at improving cognitive and motor functions through external stimulation and help to a certain extent. Each training should be maintained between 25 and 35 min, once a day, and five times a week, and continuous treatment should be provided for 8 weeks.

#### 2.3.3. hCPM group

The children received the following goal-directed training plan for 8 weeks (14): physical therapy, hand function therapy, speech therapy, guided educational therapy, and physical factor therapy (once a day, five times a week, between 25 and 35 min each time for 8 weeks). They also received continuous passive motion training of the hip joint simultaneously (5 days a week, two times a day, one for both sides of the limbs in the same direction and one for the opposite direction, with each training duration lasting between 40 and 60 min).

**Figure 2:**
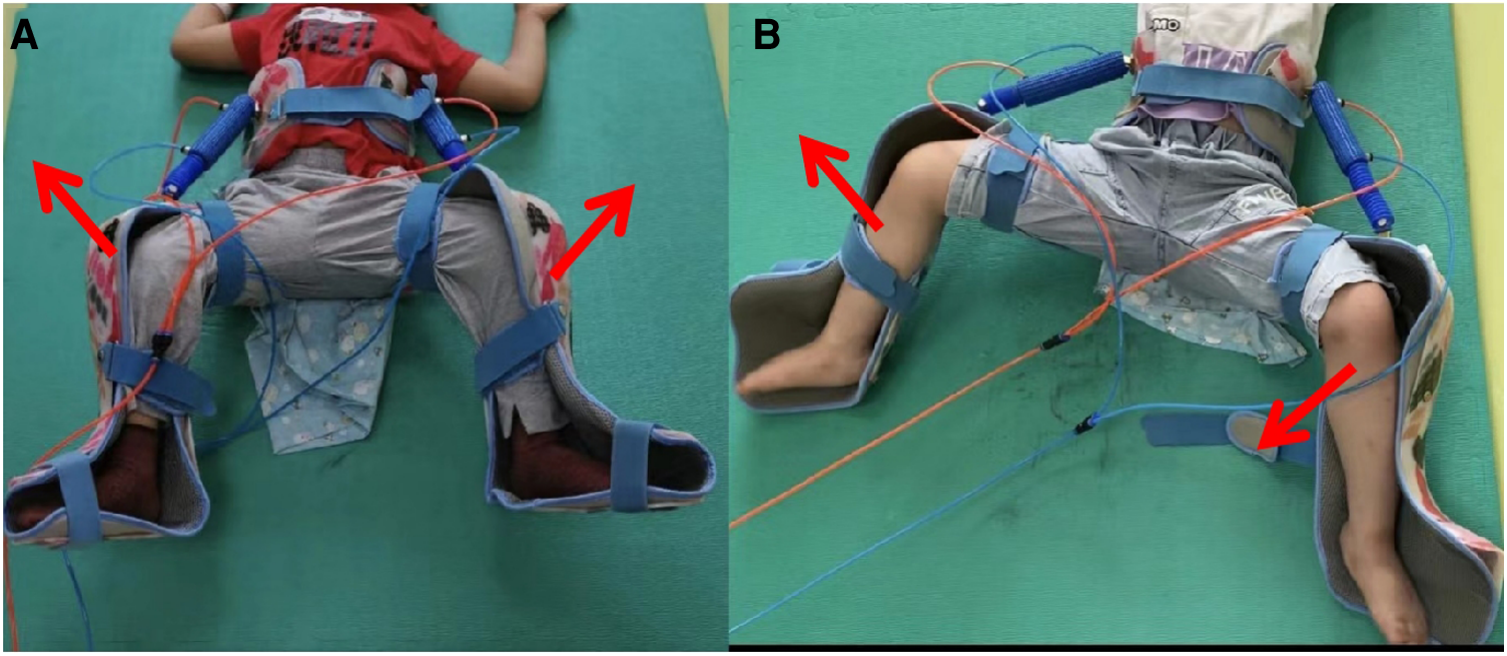
A child in hCPM group was undergoing hip continuous passive motion (Figure 2A was codirection hCPM therapy and Figure 2B was interaction hCPM therapy).

(1) First, the children’s lower limbs and pelvis were fitted with appropriate external fixators, and they were placed on flat surfaces at room temperature while the braces were also donned.
(2) The working pressure and exercise frequency were set in accordance with the child’s weight and condition, ranging from 200 to 350 kPa and 10–30 s, respectively.
(3) Codirection hCPM therapy: This therapy sets the instrument’s working mode to codirection movement. The 20 min passive up and down activity uses the hip joint as the fulcrum, with both lower limbs in the external rotation and abduction positions (Figure 2A).
(4) Interaction hCPM therapy: In this therapy, the device’s working mode is switched to interaction movement. Both lower limbs are in abduction and external rotation with the hip joint serving as the fulcrum, and both lower limbs are alternately moved up and down passively for 20 min (Figure 2B).

### 2.4. Outcome measures

#### 2.4.1. Gross motor function measure

Gross Motion Function Measure (GMFM-88) (15, 16) was used to evaluate the gross motion function of the children before and after 8 weeks of treatment, which is divided into five functional areas. There were a total of 88 items, including the decubitus position and turning over, sitting position, crawling and turning over, standing, walking and running, and jumping, and the evaluation results included each ability, total score, and total percentage. The total percentage was used for evaluation in this study.

#### 2.4.2. MP and AI value

**Figure 3:**
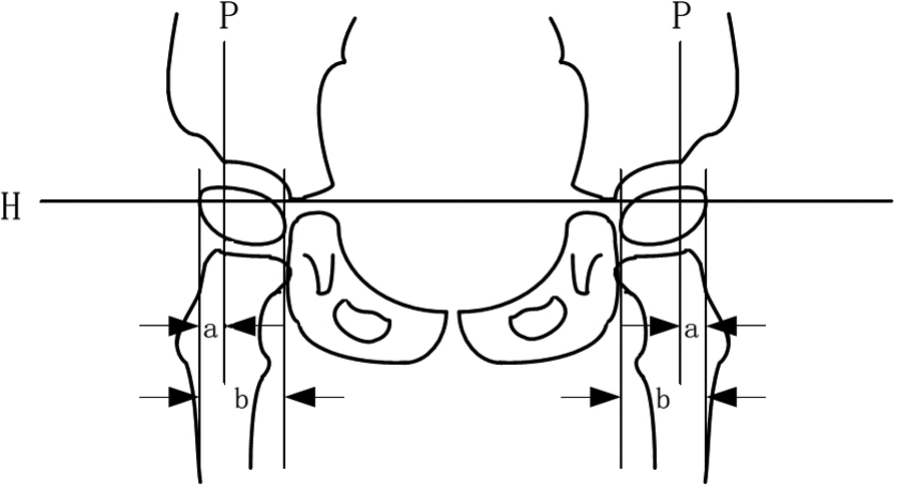
Calculation methods of MP and AI values in plain hip x-ray films. MP, migration percentage; AI, acetabular index.

The structure and function of hip joint development in enrolled children were respectively evaluated by a practicing evaluator who has received more than half a year of professional training and work experience before 8 weeks of treatment: bilateral hip x-ray plain films migration percentage (MP), acetabulum index (AI), and Harris hip score before and after the change (see Figure 3).

The acetabulum is composed of the ilium, pubis, and ischium, and the three bones are joined together by a y-shaped cartilage, which is closed when the child attains approximately 13 years of age. The apex of the y-shaped cartilage is the center of the acetabulum, and the lowest point of the ilium on radiographs is often referred to as the apex of the y-shaped cartilage.

#### 2.4.3. Migration percentage

A line was made through the vertex of the inner and lower margins of the two parts of the acetabulum (*H*), the outer and upper margins of the acetabulum were used as a vertical line (*P*), and the ratio of the outer femoral head part (*A*) of the *P* line to the femoral head transverse diameter (*B*) was multiplied by 100% (17–19), that is, MP = *A*/*B* × 100%.

The clinical grade and significance of the MP value: an MP value>33% signifies 100 points for subluxation of the hip joint, and 50% denotes total dislocation of the hip joint. The other grades are as follows: Grade I, slight displacement: MP < 25%; Grade II, risk: MP = 25%–32.9%; Grade III, subluxation: MP = 33%–49.9%; Grade IV, severe subluxation: MP = 50%–89.9%; Grade V, total dislocation: MP > 90%.

#### 2.4.4. Acetabular index

A straight line is drawn through the vertex of the y-shaped cartilage of the acetabulum on both sides and it is extended. Then, a straight line is connected from the vertex of the y-shaped cartilage to the most prominent point of the lateral upper edge of the top of the osseous acetabulum, and the sharp angle between the two is the acetabular index (18).

Clinical significance of the AI value: The acetabular index of a newborn should not be more than 30°, approximately 25° at 1 year of age, approximately 20° at 2 years, less than 20° at 2 years’ old and more, and approximately 10° in adults. Generally, acetabular dysplasia or dislocation occurs when the index is above 30°. A degree greater than this indicates acetabular dysplasia, and the acetabular index is often higher in children with cerebral palsy.

#### 2.4.5. Harris hip function score

The Harris hip function score (HHS was used to evaluate the hip functions of the children such as pain, daily life and gait function, range of motion, and deformity. The full score was 100, and the higher the score, the better will be the function (20).

Clinical significance of Harris score: a full score of 100; <70 indicates poor function, a range of 70–79 indicates fair function, 80–89 indicates good function, and >90 indicates excellent function.

### 2.5. Statistical analysis

All statistical analyses were conducted by using the difference test, and a *P-*value less than or equal to 0.05 means that the difference is statistically significant. The Chi-square test was used to compare the rates of the two groups, and the measurement data were statistically described using mean ± SD. Unlike during the screening period when the baseline value was used, an independent sample *t*-test and a paired sample *t*-test were used to compare the differences of intra-group and inter-group and between the hCPM group and the control group.

## 3. Results

A total of 65 children who met the inclusion criteria were included in the study, out of which 45 patients were included in the hCPM group, which comprised 28 males and 17 females aged (46.20 ± 17.09) months. The remaining 20 patients were included in the control group, which comprised 14 males and 6 females aged (37.85 ± 14.90) months. A comparison of the mean and SD of the sex and age of the two groups showed that the difference was not statistically significant (*P* ≥ 0.05, *P* values were 0.545 and 0.064, respectively), as shown in Table 1.

**Table 1:** Baseline characteristics of participants who enrolled in an 8-week single-center randomized controlled study.

		hCPM group	Control group	*χ*²/*t*	*P*
Gender (*n*)	Male	28	14	0.366	0.545
	Female	17	6		
Age (month)		46.20 ± 17.09	37.85 ± 14.90	1.886	0.064
GMFCS	III	29	12	0.117	0.732
	IV	16	8		
	Nil aid	3	6		
	Walking frame	16	4		
Primary mobility aid (*n*)	Manual wheelchair (self-propels)	18	5		
	Manual wheelchair (does not self-propel)	4	3		
	Power wheelchair	4	2		

hCPM, hip continuous passive motion.

We also included the GMFCS grading data of children in each group and compared the cerebral palsy grading of children participating in the study. Among them, there were 29 patients with a GMFCS grading of level III and 16 patients with a GMFCS grading of level IV in the hCPM group. There were 12 patients with a level III grading and 8 patients with a level IV grading in the control group. There was no significant difference in the mean and SD of GMFCS grading between the two groups (*P* ≥ 0.05, and the *P*-value was 0.732).

In addition, the main assistive devices used by children in each group during walking were counted. In the hCPM group, there were 3 children who did not need any assistance to walk, 16 children needed assistance from others, 18 children used autonomous manual wheelchairs, 4 required assistance from others, and 4 others needed assistance in the form of electric wheelchairs. In the control group, there were six children who did not require any assistance to walk, four required assistance from others, five used autonomous manual wheelchairs, three needed assistance from others, and two required an electric wheelchair, as shown in Table 1.

### 3.1. Gross motor function measure

The following is a comparison of the GMFM score between the hCPM group and the control group: Before the training was provided, there was no significant difference in the mean total percentage of GMFM between the hCPM group and the control group (*t* = −1.720, *P* > 0.05, with a *P*-value of 0.090). After 8 weeks of treatment, the mean total percentage of GMFM in the hCPM group was significantly higher than that in the control group. It can be concluded that the difference was significant (*t* = −2.637, *P* < 0.05, and the *P*-value was 0.011). The following is an intragroup comparison of the GMFM score between the two groups both after and before treatment: After 8 weeks of treatment, the total percentage of GMFM in the two groups was higher than that before treatment (*t* = 18.593 and 11.430, *P* < 0.001). The results are provided in Table 2.

**Table 2:** Means (SE) of GMFM, MP, AI, and HHS at each time-point, and statistical comparison (independent and paired sample t-test).

	Prior treatment	Post-treatment	*t*	*P*
GMFM				
hCPM group	46.95 ± 22.11	58.79 ± 20.92	18.593	<0.001
Control group	36.17 ± 25.94	42.49 ± 27.22	11.430	<0.001
*t*	−1.720	−2.637		
*P*	0.090	0.011		
LEFT MP				
hCPM group	31.69 ± 2.30	27.32 ± 2.54	20.172	<0.001
Control group	30.42 ± 3.13	29.55 ± 3.41	7.065	<0.001
*T*	−1.836	2.6154		
*P*	0.071	0.014		
RIGHT MP				
hCPM group	31.91 ± 2.47	27.70 ± 2.16	40.291	<0.001
Control group	30.75 ± 3.02	29.92 ± 2.98	21.163	<0.001
*t*	−1.517	3.000		
*P*	0.139	0.06		
LEFT AI				
hCPM group	32.26 ± 4.24	28.01 ± 3.92	16.820	<0.001
Control group	30.83 ± 3.53	30.11 ± 3.54	15.176	<0.001
*t*	−1.310	2.055		
*P*	0.195	0.044		
RIGHT AI				
hCPM group	32.16 ± 4.12	27.51 ± 3.80	32.900	<0.001
Control group	30.37 ± 3.60	29.76 ± 3.64	18.834	<0.001
*t*	−1.684	2.223		
*P*	0.097	0.030		
HHS				
hCPM group	57.60 ± 12.20	76.67 ± 9.25	28.081	<0.001
Control group	53.90 ± 15.29	58.80 ± 15.90	9.759	<0.001
*t*	−1.042	−4.685		
*P*	0.301	0.000		

hCPM, hip continuous passive motion; GMFM, gross motor function measure; MP, migration percentage; AI, acetabular index; HHS, Harris hip functional score.

### 3.2. Hip structural assessments

#### 3.2.1. Intragroup comparison of the LEFT MP value between the two groups

Before treatment, the mean ± SD of the left MP value of the hCPM group was 31.69 ± 2.30. After 8 weeks of treatment, the mean ± SD was 27.32 ± 2.54. It can be concluded that the mean value after treatment was higher than that before treatment (*t* = 20.172, *P* < 0.001), and the difference was statistically significant.

Before treatment, the mean ± SD of the left MP value in the control group was 30.42 ± 3.13, and after 8 weeks of treatment, the mean ± SD was 29.55 ± 3.41. It can be concluded that the mean value after treatment was higher than that before treatment (*t* = 7.065, *P* < 0.001), and the difference was statistically significant. The results are given in Table 2.

#### 3.2.2. Intragroup comparison of the RIGHT MP value between the two groups

Before treatment, the mean ± SD of the right MP value of the hCPM group was 31.91 ± 2.47, and after 8 weeks of treatment, the mean ± SD was 27.70 ± 2.16. It can be concluded that the mean value after treatment was higher than that before treatment (*t* = 40.291, *P* < 0.001), and the difference was statistically significant.

Before treatment, the mean ± SD of the right MP value in the control group was 30.75 ± 3.02, and after 8 weeks of treatment, the mean ± SD was 29.92 ± 2.98. It can also be concluded that the mean value after treatment was higher than that before treatment (*t* = 21.163, *P* < 0.001), and the difference was statistically significant. The results are provided in Table 2.

#### 3.2.3. Intragroup comparison of the LEFT AI value between the two groups

Before treatment, the mean ± SD of the left AI value of the hCPM group was 32.26 ± 4.24, and after 8 weeks of treatment, the mean ± SD was 28.01 ± 3.92. It can be concluded that the mean value after treatment was higher than that before treatment (*t* = 16.820, *P* < 0.001), and the difference was statistically significant.

Before treatment, the mean ± SD of the left AI value of the control group was 30.83 ± 3.53, and after 8 weeks of treatment, the mean ± SD was 30.11 ± 3.54. It also can be concluded that the mean value after treatment was higher than that before treatment (*t* = 15.176, *P* < 0.001), and the difference was statistically significant. The results are given in Table 2.

#### 3.2.4. Intragroup comparison of the RIGHT AI value between the two groups

Before treatment, the mean ± SD of the right AI value of the hCPM group was 32.16 ± 4.12, and after 8 weeks of treatment, the mean ± SD was 30.11 ± 3.54. It can be concluded that the mean value after treatment was higher than that before treatment (*t* = 32.900, *P* < 0.001), and the difference was statistically significant.

Before treatment, the mean ± SD of the right AI value of the control group was 30.83 ± 3.53. After 8 weeks of treatment, the mean ± SD was 30.11 ± 3.54. It also can be concluded that the mean value after treatment was higher than that before treatment (*t* = 18.834, *P* < 0.001), and the difference was statistically significant. The results are provided in Table 2.

#### 3.2.5. Intergroup comparison of the LEFT MP value between the two groups

Before treatment, the mean ± SD of the left MP value in the hCPM group was 31.69 ± 2.30, and the mean ± SD in the control group was 36.17 ± 25.94. There was no significant difference between the two longitudinal groups (*t* = −1.836, *P* > 0.05, with a *P*-value of 0.071).

However, after 8 weeks of treatment, the mean ± SD of the left MP value in the hCPM group was 27.32 ± 2.54, and the mean ± SD in the control group was 29.55 ± 3.41. The longitudinal comparison between the two groups showed that the difference was significant (*t* = 2.615, *P* < 0.05, with a *P*-value of 0.014). The results are provided in Table 2.

#### 3.2.6. Intergroup comparison of the RIGHT MP value between the two groups

Before treatment, the mean ± SD of the right MP value in the hCPM group was 27.70 ± 2.16, and the mean ± SD in the control group was 30.75 ± 3.02. There was no significant difference between the two longitudinal groups (*t* = −1.517, *P* > 0.05, with a *P*-value of 0.139).

However, after 8 weeks of treatment, the mean ± SD of the right MP value in the hCPM group was 27.32 ± 2.54, and the mean ± SD in the control group was 30.11 ± 3.54. The longitudinal comparison between the two groups showed that the difference was significant (*t* = 3.000, *P* < 0.05, and the *P*-value was 0.039).The results are provided in Table 2.

#### 3.2.7. Intergroup comparison of the LEFT AI value between the two groups

Before treatment, the mean ± SD of the left AI value in the hCPM group was 32.26 ± 4.24, and the mean ± SD in the control group was 30.83 ± 3.53. There was no significant difference between the two longitudinal groups (*t* = −1.310, *P* > 0.05, with a *P*-value was 0.195).

However, after 8 weeks of treatment, the mean ± SD of the left AI value in the hCPM group was 28.01 ± 3.92, and the mean ± SD in the control group was 29.55 ± 3.41. The longitudinal comparison between the two groups showed that the difference was significant (*t* = 2.055, *P* < 0.05, with a *P*-value of 0.044).The results are given in Table 2.

#### 3.2.8 Intergroup comparison of the RIGHT AI value between the two groups

Before treatment, the mean ± SD of the right AI value in the hCPM group was 32.16 ± 4.12, and the mean ± SD in the control group was 30.37 ± 3.60. There was no significant difference between the two longitudinal groups (*t* = −1.684, *P* > 0.05, with a *P*-value of 0.097).

However, after 8 weeks of treatment, the mean ± SD of the right AI value in the hCPM group was 27.51 ± 3.80, and the mean ± SD in the control group was 58.80 ± 15.90. The longitudinal comparison between the two groups showed that the difference was significant (*t* = 2.223, *P* < 0.05, with a *P*-value of 0.030). The results are provided in Table 2.

### 3.3. Hip functional assessments

#### 3.3.1. Intragroup comparison of HHS between the two groups

Before treatment, the mean ± SD of the Hip Harris Function score of the hCPM group was 57.60 ± 12.20. After 8 weeks of treatment, the mean ± SD was 76.67 ± 9.25. It can be concluded that the mean value after treatment was higher than the mean value before treatment (*t* = 28.081, *P* < 0.001), and the difference was statistically significant.

Before treatment, the mean ± SD of the Hip Harris Function score in the control group was 53.90 ± 15.29. After 8 weeks of treatment, the mean ± SD was 58.80 ± 15.90. It can be concluded that the mean value after treatment was higher than that before treatment (*t* = 9.759, *P* < 0.001), and the difference was statistically significant. The results are presented in Table 2.

#### 3.3.2. Intergroup comparison of HHS between the two groups

Before treatment, the mean ± SD of the Hip Harris Function score in the hCPM group was 57.60 ± 12.20, and the mean ± SD in the control group was 53.90 ± 15.29. There was no significant difference between the two longitudinal groups (*t* = −1.042, *P* > 0.05, with a *P*-value of 0.301).

However, after 8 weeks of treatment, the mean ± SD of the Hip Harris Function score in the hCPM group was 76.67 ± 9.25, and the mean ± SD in the control group was 58.80 ± 15.90. The longitudinal comparison between the two groups showed that there was a significant difference (*t* = −4.685, *P* < 0.05, with a *P-*value of 0.000). The results are given in Table 2.

## 4. Discussion

### 4.1. Musculoskeletal development disorder in cerebral palsy

Cerebral palsy belongs to a group of nervous system diseases caused by continuous movement and posture disorders caused by abnormal brain development (8, 21, 22). It is often accompanied by epilepsy, skeletal muscle deformity, sensory perception, visual perception, and cognitive problems. At present, the specific cause of the disease is still unknown. It may be caused by a series of complex factors such as heredity, prenatal factors (such as hypoxia and ischemia, intrauterine infection, or growth restriction), and non-permanent labor (23–25). However, in approximately 80% of the cases, the cause of the disease cannot be determined, and therefore, some people opine that the disease is idiopathic (2).

Among all children with CP, one-third of them have hip displacement, which ranks as the second most common musculoskeletal deformity, second only to horseshoe varus and valgus. Research review results also indicate that hip displacement (26, 27) is directly related to the gross motor function determined by the GMFCS of cerebral palsy (28).

The incidence rate among children with osteoarticular dysplasia who show hip dysplasia and hip dislocation symptoms ranges between 27% and 35% (29–31). At the same time, there are symptoms of lower limb dysfunction, impaired balance sitting posture, perineal nursing issues, and bedsores.

The entry point of this study is the common orthopedic problems found in children with cerebral palsy, for example, the musculoskeletal dysplasia of the hip joint, including acetabular dysplasia, subluxation, and total dislocation of the hip joint. The selected subjects are 117 children diagnosed as spastic cerebral palsy at the Children's Neurological Rehabilitation Center of the High-tech Zone of our hospital, and the target group consists of 80 children with a dysplasia of the hip joint. Approximately 68.3% of children with cerebral palsy have problems related to the development of the hip joint. Such musculoskeletal development disorders may cause obvious pain and motor dysfunction in children.

This study found that the risk of hip dislocation in children with spastic cerebral palsy was higher than that of other types of cerebral palsy, and it was related to the degree of motor dysfunction. The motor development of children with cerebral palsy generally lags behind that of normal children of the same age. They start walking late, have poor walking ability, and lack the opportunity to bear weight in their lower limbs, all of which aggravates the outward displacement of the femoral head. At the same time, the ligaments and muscles that fix the acetabulum are not fully exercised, and the fixation of the femoral head is insufficient, resulting in changes in the shape of the hip joint and the position of the femoral head. On the other hand, due to the uncoordinated control of muscles around the hip joint, the muscle tension of the adductor femoris increased and remained for a long time, while the muscle strength and tension of the gluteus medius and iliopsoas muscles were relatively low, which caused a further adduction of the hip joint, and the femoral head was pulled outward. With the extension of time, there was a gradual subluxation of the hip joint. The more serious the condition of children with cerebral palsy is, the higher the incidence of subluxation of the hip joint. If it is not treated in time and also effectively, it will eventually lead to a total dislocation of the hip joint.

### 4.2. Developmental characteristics and countermeasures of the hip joint in children with CP

The abnormality of bone and joint development has gradually become the research hot spot in children with cerebral palsy, and the rate of incidence of hip dysplasia in children with moderate and severe cerebral palsy is increasing. How to precisely monitor and correct this kind of deformity has become a focus point of research calling for urgent attention.

When a child with cerebral palsy is born, most of the hip joint structures that can be monitored on the two-dimensional plane are normal, not half dislocated or not fully dislocated. However, with the development of the basic disease of cerebral palsy, a stiffness or shortening of the soft tissues around the hip joint, such as the muscles, ligaments, and joint capsule. It will lead to a chronic abnormal static posture and result in contracture. With the adductor and hip flexor being pulled abnormally strongly or spasmodically, it will lead to femoral pronation flexion and adduction, in an abnormal position of the femoral head and acetabulum, even in an abnormal shape of the acetabulum (30, 31). A large number of studies in the literature have discussed the natural evolution of hip dysplasia, and its etiology is quite clear, but the relative importance of different factors remains controversial to this day.

What steps should be taken to reduce the incidence of long-term serious sequelae caused by the dislocation of the hip joint? The right countermeasure is to conduct a regular and periodic radiological x-ray examination on the children to determine the spatial position relationship between the two parts of the acetabulum and the femoral head (7).

Picciolini et al. (32) emphasized the importance of early detection of high-risk hip joints and the possibility of implementing early hip joint prevention programs in children with cerebral palsy. If the MP value of children with cerebral palsy is within the normal range (i.e., <21%) at the beginning of treatment, it can be predicted that their MP value will remain within the normal range during later growth and development. In contrast, if the MP value shifts to a certain extent outside the normal range during early monitoring, the value will gradually increase over time from 23.0% to 37.7% after 2 years. Other studies have shown that the MP index increases by 7.7% annually in non-walking patients and by 4% annually in those who may walk.

Particularly for children of grade IV and V in the GMFCS, different treatment methods should be selected according to their clinical and functional status, MP value, and long-term prognosis to achieve the best effect. If the MP index is between 30% and 50%, soft tissue surgery can effectively balance the muscle strength of the entire hip joint.

However, with the gradual decline of gross motor function, the quality of life of children plummets, and therefore, orthopedic surgery is suggested as the intervention (33). Multilevel surgery or single multilevel surgery currently involves more than four orthopedic operations for multiple joints of both lower limbs at the same time. Along with postoperative rehabilitation, it is currently a common treatment method for spastic diplegia and quadriplegia with walking ability (34). However, in this study, the follow-up period of 5–10 years after operation showed that the gait, independence, and self-satisfaction of children with cerebral palsy had improved slightly, but the improvement in gross motor function was not significant.

Therefore, in order to reduce the progression of hip joint dislocation in these children with spastic cerebral palsy to the need for surgical intervention and also to reduce the rate of operation, this study started from the point of non-surgical treatment and made best use of the advantage of the continuous operation offered by external instruments to observe whether the gross motor function and the development of the hip joint could be improved.

### 4.3. The principle and significance of CPM in the treatment of hip joint problems in children with CP

Based on the original intention of making every effort to improve these problems, the research team drew on the theoretical basis and wisdom of its predecessors, and based on Wolf’s law and Ilizarov’s tensile stress law, designed a continuous passive motion instrument for the hip joint. As is known, Wolfe's law is the adaptive principle of the bone, and Ilizarov’s tension stress law is also known as traction osteogenesis technology or traction tissue regeneration technology (35). This kind of device makes use of external force to make the subject’s hip joint carry out a series of uniform, continuous, uniform, and slow passive movements, so as to improve the blood circulation and nerve function in this area. This improvement in the excitability of nerve activity can lead to a benign circulation and promote the recovery of the skeletal muscle to its normal condition.

After a great deal of research, Knapik et al. proposed that the mechanical signals generated by continuous passive movement may be perceived by mechanically sensitive chondrocytes in the articular cartilage, which can help stabilize the internal environment and organizational structure of the articular cartilage, reduce joint stiffness, reduce the possibility of complications related to adhesion, promote the generation of new cartilage, form and preserve normal articular cartilage, effectively prevent the stiffness and adhesion of the fibrous tissue, significantly improve the health of chondrocytes and the recovery of the normal joint tissue, and finally help retain the appearance of the normal joint cartilage. Compared with fixed and intermittent active movement, it has significantly better kinematic, histological, and biological characteristics (36).

Compared with traditional fixation and support, CPM takes advantage of its dynamic stability, that is, the subject’s hip joint can perform passive abduction and adduction movement within the safe angle range, so that the acetabulum and femoral head are constantly stimulated by each other. This benign contact can promote the continuous circulation of a joint fluid in the hip joint, thus improving the nutritional status of the articular cartilage and promoting the further development of the hip joint. Preventing its further development to dislocation can theoretically improve the rehabilitation effect of the hip joint in children with cerebral palsy. Compared with the previous conservative rehabilitation treatment, this technology greatly reduces the time and energy required for artificial fixation of the hip joint, thereby reducing the incidence of femoral head necrosis and hip joint motion limitation.

Based on the superior histological effect seen in the continuous passive movement therapy, CPM has been proved to be a useful auxiliary intervention treatment strategy. It is necessary to carry out further research in this field to explore the importance of the starting time of CPM therapy and clearly indicate that the best time, duration, and intensity of starting the therapy will continue to be the focus of future research.

### 4.4. The significance of the study

This study was a prospective randomized controlled one. A total of 65 children were included in the study and randomly divided into two groups, namely, a CPM instrument treatment group (45 cases) and a conventional treatment group (20 cases). Structural data (MP value and AI value) and HHS were obtained by using the gross motor function scale (GMFM-88) and a bilateral hip joint plain film. The aim was to explore the curative effect of treating spastic cerebral palsy children with a dysplasia of the hip joint by a continuous passive activity of the hip joint.

The imaging measurements currently used to evaluate the hip joint of neuromuscular dysplasia of the hip include the percentage of femoral head migration (MP value) and the AI value. The percentage of femoral head deviation is a continuous variable used to measure the percentage of the femoral head to the outside of the acetabular edge. The reliability and repeatability of using MP and AI have been investigated and found to be acceptable, with an error percentage in the range of 6–13% (37). Therefore, this study uses the MP value and AI value as the data to evaluate the hip joint structure, so that it is more representative and convincing in nature.

The research results showed that after the continuous passive activity of the hip joint for 2 months, the CPM instrument treatment group showed significant differences in terms of the improvement in the total percentage of the GMFM score, bilateral MP value, AI value, and HHS score, compared with the conventional treatment group, indicating that the use of the CPM instrument of the hip joint for intervention training on the basis of the conventional rehabilitation treatment can significantly improve the gross motor function, the structure and function of the hip joint, and the rehabilitation effect of such children.

At the same time, an analysis of the research data of the routine treatment group shows that even without the intervention training of CPM, the continuous routine rehabilitation for a short duration can still improve the gross motor function and the structure and function of the hip joint to a certain extent, which further confirms the necessity of timely rehabilitation treatment for children with cerebral palsy.

Therefore, the continuous passive activity of the hip joint proves that it can promote the formation of healthier cartilage, which is located closer to the original articular cartilage. By making the fluid in the joint flow to the compartment outside the joint capsule, it may speed up the removal of harmful stimulation factors and prevent the rapid degradation of cartilage, leading to an improvement in the clinical results. Functionally, the production of healthier cartilage with less exposure to inflammatory molecules may help improve the prognosis of children with spastic cerebral palsy and reduce the rates of occurrence and development of hip dislocation in such children.

### 4.5. Adverse reactions

During the implementation of the clinical trial intervention, adverse reactions are common. During the 2-month rehabilitation training conducted in this study, four patients developed complications with severe infection during the treatment, and one patient suffered from a partial necrosis of the femoral head. These five patients were unable to continue participating in this study because of the complications that arose from such infections. However, the remaining 65 children were able to cooperate well with the rehabilitation training. Sometimes, even upper respiratory tract infection or mild digestive tract infection will occur occasionally during the treatment process and the patients will need rest for 1–2 days to recover. But, this break in training time can be compensated during the weekend or later during the course of the study period. Such mild adverse reactions did not have a great negative impact on our research, which proved that continuous passive activity training of the hip joint was safe and effective, and is, therefore, worthy of clinical promotion.

### 4.6. Limitations of the study

The sample size included in this study is not large and therefore may not comprehensively cover the actual and entire treatment process. The study subject is a child with spastic cerebral palsy diagnosed as having a dysplasia of the hip joint, but this cannot represent all types of children with cerebral palsy, and therefore, this a limitation. A delay occurred during the 8-week course of treatment because a few of the study subjects developed a mild upper respiratory tract infection or digestive tract infection during this period; however, all of them made up for the lost training time soon after their recovery. It is possible that the specified 2 months of continuous passive activities have not been strictly achieved to.

This study has completed only 2 months of rehabilitation training, which indicates that it is an early stage and a relatively short period of the complete treatment cycle. Also, it has not carried out a long-term follow-up evaluation post-treatment, and only a rehabilitation evaluation has been carried out for the current post-treatment period. Therefore, it can be concluded that it is necessary to carry out a large-sample randomized controlled study to extend the treatment time and also a long-term follow-up evaluation at the end of the treatment. But a multicenter clinical research is required to achieve these objectives. The ultimate objective should be to provide a more powerful evidence-based medical basis for CPM treatment of dysplasia of the hip and to explore the effectiveness and actual benefits of this hip CPM instrument on children with spastic cerebral palsy.

## 5. Conclusion

In this prospective study, after a short-term intensive target-oriented exercise rehabilitation training for children with spastic cerebral palsy, it can be observed that the gross motor function of these children and the structure and function score of the hip joint improve significantly; this improvement effect is more obvious after the training is combined with the continuous passive activity of the hip joint. Apart from some initial maladaptations, no other adverse reactions were observed, indicating that the rehabilitation program has good safety and efficacy. However, this result may also be related to the limited observation time and the small number of samples in this study. The study was carried out only in the children’s rehabilitation center of our hospital. Therefore, more multicenter and large-sample cohort clinical trials are needed to provide a more powerful evidence-based clinical basis for determining the optimal time, duration, and intensity of CPM treatment. And use it as a basis to figure out the optimal intensity and duration of training to obtain the best effect. So that more children can retain the most basic motor ability, walk up and more smoothly enter and adapt to society.

## Data Availability

The raw data supporting the conclusions of this article will be made available by the authors, without undue reservation.
